# Using Ecological Diversity Analyses to Characterize the Availability of Healthy Food and Socio-Economic Food Deserts

**DOI:** 10.3390/ijerph181910297

**Published:** 2021-09-29

**Authors:** Annie Goyanes, Jeffrey Matthew Hoch

**Affiliations:** Department of Marine and Environmental Sciences, Nova Southeastern University, 3301 College Ave, Fort Lauderdale, FL 33314, USA; ag2484@mynsu.nova.edu

**Keywords:** food desert, supermarkets, social justice, food supply

## Abstract

“Food deserts” are usually defined as geographic areas without local access to fresh, healthy food. We used community ecology statistics in supermarkets to quantify the availability of healthy food and to potentially identify food deserts as areas without a diverse selection of food, rather than a binary as to whether fresh food is present or not. We test whether produce diversity is correlated with neighborhood income or demographics. Abundance and diversity of fresh produce was quantified in supermarkets in Broward County, Florida, USA. Neighborhood income level and racial/ethnic makeup were retrieved from the U.S. Census and American Community Survey. Although diversity varied, there were no communities that had consistently less available fresh food, although the percent of a neighborhood identifying as “white” was positively correlated with produce diversity. There may be fewer choices in neighborhoods with a higher proportion of minorities, but there were no consistent patterns of produce diversity in Broward County. This method demonstrates an easy, inexpensive way to characterize food deserts beyond simple distance, and results in precise enough information to identify gaps in the availability of healthy foods.

## 1. Introduction

Food deserts, defined by the U.S. Department of Agriculture, are urban neighborhoods and rural towns without ready access to fresh, healthy, and affordable food and are defined by the distance that people must travel to access grocery stores, supermarkets or other food stores [1]. Food deserts often are in low-income, predominantly minority neighborhoods [2]. These neighborhoods, compared to higher-income neighborhoods, have fewer fruit and vegetable markets and bakeries but may have an abundance of liquor stores, smaller grocery stores, and meat and fish markets [3]. Distance to supermarkets is often greater for people who live in low-income neighborhoods [3] and lack of public or private transportation creates another barrier that contributes to food poverty (a lack of access to healthy foods). Besides distance, urban residents’ shopping decisions are influenced by the characteristics of food retailers, residential segregation, and perceptions of neighborhood safety [4]. Supermarket redlining is when major chain supermarkets are reluctant to locate stores in low-income areas and instead stores are placed in the suburbs. Thus, food options, price, and quality can vary due to the differing socioeconomic neighborhoods [2].

Food deserts are typically measured and identified by measuring physical distance and economic access (the relative price of fresh produce items and the cost of transportation to acquire them) [5]. The physical distance between neighborhoods and grocery stores are measured between census tracts or by referencing 1-square-kilometer grids for geographical analysis. For each grid cell, the distance from the nearest grocery store is used to measure access for people who live in that grid. Then, the analysis of sources of consistent healthy food by measured through various analytical tools [5]. Food deserts have also been characterized using other measures of physical distance and geographical distribution of food sources [6,7,8], using geographic information systems and geographic accessibility modeling [9]. Food availability within this geographic context has been quantified using the square footage of the fruit and vegetable section of markets [10], the linear shelf space devoted to fresh produce [8], surveys of food availability and whether those foods meet healthy diet guidelines [11] and discrepancy between local supply and demand, based on local cultural preferences [12]. Despite this work, few studies have quantified relative levels of fresh, healthy food products within neighborhoods with differing ethnic or economic makeups with respect to food deserts.

In the 1960s, white, middle-class families left for suburbs and the supermarkets followed and started developing chains. In the 1980s, the food retail industry in the U.S. had a pronounced shift from large supermarkets to superstores [13]. The stores not only offered food items, but non-food items had a wide diverse presence in these superstores. The biggest influence for large markets was the growth of Walmart Supercenters. The Walmarts started attracting frequent shoppers and had greater profits for it, which pressured other food retailers to expand. Urban development encouraged these stores, additionally the shoppers were attracted to the lower prices, wider selection, and easier parking. These large supermarkets started to contribute to food deserts because of the seemingly unequal physical distribution of food retailers [13]. The history of supermarkets developing in the U.S. has also contributed to present-day supermarkets distributions and the development of food deserts [13].

All these factors play into the environmental or economic injustice that can occur in these low-income neighborhoods. Decreased access to healthy food means people in low-income communities suffer more from diet-related diseases like obesity and diabetes than those in higher-income areas. Access to healthy food is associated with lower risk of obesity and other diet-related chronic diseases [14].

South Florida has been described as a microcosm of the United States, in terms of diversity and health disparities and is thus an ideal place to investigate the dynamics of food deserts [15]. South Florida’s population is increasingly hungry with a food insecurity percentage of 14.3% in Broward County. In South Florida, 18.9% of children are food insecure thus not being able to properly develop and succeed in school [16]. In Florida, 11.7% of households were unable to provide adequate food for one or more household members [17]. Florida’s food insecurity rate is like the overall food insecurity rate in U.S. households: 11.1% [18]. The presence of socioeconomic food deserts may be one of the factors of Broward’s high food insecurity rate.

We propose that the environment of the supermarket is analogous to an ecological community, where diversity can be measured, similarity between environments quantified and missing elements identified. The goal of this research is to use ecological statistics to determine whether the socioeconomic stratification in Broward County, FL, USA, is associated with the diversity of produce available in stores from different neighborhoods. We tested the null hypothesis that the diversity of products available at supermarkets in Broward do not vary with respect to socioeconomic, ethnic or racial makeup of the neighborhood. The alternative hypothesis is that product diversity is correlated to socioeconomic, ethnic or racial makeup of neighborhoods. We tested this by investigating whether the assemblages of fresh produce products were similar in supermarkets found in neighborhoods of different socioeconomic makeup. We also tested whether wealthy, predominantly white neighborhoods had similar assemblages of produce to poorer neighborhoods or neighborhoods with a higher proportion of other races. At the same time, we also investigated differences between different markets, since availability of produce might be more associated with the individual companies than the makeup of the communities in which they reside.

## 2. Materials and Methods

### 2.1. Data Collection

**Figure 1:**
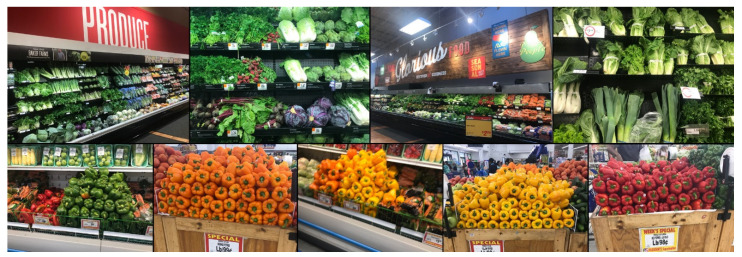
Representative images from supermarkets. Top row: Representative pictures of Supermarket #2 in District 98 (first), Superstore #1 in District 97 (second), Lucky Market in District 98 (third), and Save A Lot, District 94 (fourth). Bottom row: Representative pictures of Presidente in District 102, an independent store. This store had an increased abundance of various types of bell peppers.

Every Thursday afternoon between 12 September 2019 and 28 November 2019, three markets in each of the 13 Florida House of Representatives Districts in Broward County were examined. Florida State House Districts were used to fully explore the diversity of neighborhood ethnicity, social class and racial make-up, based on the assumption that political districts are gerrymandered to ensure representation by distinct groups and to ensure we sampled the complete range of communities in Broward County (gerrymandering refers to the intentional demarcation of political districts, ostensibly done to ensure fair representation of all communities, but often it leads to unfair political advantage by the ruling political party) [19]. It also ensured that our sampling was well dispersed geographically. Each grocery store for the week was in the same State House District, but different census tracts. The census statistical atlas was used to determine the precise locations of the boundaries of the State House Districts [20]. In each district, we attempted to sample major chain supermarkets, high-end markets focusing on fresh, organic products, and smaller, local independent markets. For each market, the unpackaged, refrigerated produce was photographed, counted, and logged (Figure 1). Demographic statistics for the neighborhood of each store, including measures of annual median income, racial/ethnic makeup, and educational attainment were collected from U.S. Census Bureau data and from the American Community Survey [21,22].

### 2.2. Data Analysis

The 39 Supermarkets were classified into five categories. “Supermarket #1” (N = 13) was Publix Super Market Inc., which sells groceries, produce and sundries at over 1200 locations in the southeastern USA. “Supermarket #2” (N = 4) was Winn-Dixie Stores Inc., which has about 500 stores in the southeastern USA. “Superstore #1” (N = 6) was Walmart Inc., a combined department store and supermarket that sells groceries, department store sundries, and general merchandise at over 10,000 locations in the USA and elsewhere (although not all of them have dedicated food, groceries, or fresh produce). The “High-end” category combined Whole Foods Market (N = 2) and The Fresh Market (N = 2), both of which specialize in organic and “natural” foods and are commonly seen as higher-end, “healthier” markets. They are generally more expensive. “Independent” markets consisted of Bravo, Festival, Food Town, Lucky Market, Presidente, Save-A-Lot and Sedanos. Only Presidente (N = 4) and Sedanos (N = 3) were represented by more than one sample. These markets are smaller chains, some of which are locally owned and operated. Our anecdotal observations were that these stores were of approximately similar size and organization. Census data [21] were used to obtain the median household income and the percent of the population identifying as “white” (calculated as 1—the total percent minority). Communities were classified by the percent of the population identifying as “White”, with “Lowest” (below 50%, N = 7), “Low” (50–70%, N = 10), “Medium” (70–80%, N = 9), “High” (80–85%, N = 7) and “Most” (85% and higher, N = 6). Median household income levels were placed into categories of “Lowest” ($19,691–$38,594, N = 7), “Low” ($42,197–$49,167, N = 11), “Medium” ($51,268–$59,291, N = 8), “High” ($65,938–$67,994, N = 4) and “Rich” ($80,865–$152,083, N = 9). All of these classifications were chosen based on existing breaks in the data and to generate groups with approximately similar sample size.

Photographs taken in the produce sections of each store were inspected and individual produce items were identified and quantified. After checking that the data met standard assumptions, we performed a three-way ANOVA using a general linear model (SAS 9.4, SAS Institute, Cary, NC, USA) to determine whether total product richness (total number of produce items available) differed significantly among store classifications, income classifications, percent white classifications and all of the two-way interactions. We did not test for the three-way interaction because our sample size resulted in too few degrees of freedom. We used a Tukey Post hoc test to determine which stores had significantly different richness. We opted not to use common diversity indices (such as the Shannon Diversity Index or the Simpson Diversity Index) because those calculations take both richness and evenness (relative proportions of each product) into account. Since we were interested in simple product availability, because some items will always be in lower demand and because we did not have access to stocking schedules, we deemed that evenness was an inappropriate measure to include in this analysis. However, such indices would be quite useful for future studies of this type and are easily calculated. To further understand the data, we performed multiple regression analysis to determine the relationship of median income and percent white with total product richness, this time using continuous variables, rather than categorical.

Primer 5.0 (Primer-E Ltd., Quest Research Limited, Auckland, New Zealand) was used to create a Bray–Curtis Dissimilarity Matrix of produce in the stores. Briefly, Bray–Curtis Dissimilarity Statistic between sites i and j can be calculated as: BC_ij_ = 1 − (2C_ij_ / [S_i_ + S_j_]), where C is the taxa in common between sites i and j and S represents taxa unique to either site [23]. The calculation is done for all possible pairs of sites, creating a matrix. We used only presence/absence data to account for disparity in restocking shelves with regularly carried items. We used the matrix to create a Multi-Dimensional Scaling (MDS) ordination of the multivariate data (with 99 restarts). The ordination produces a chart in which the relative similarity in product selection between any two points is proportional to the distance between those points. A two-way crossed Analysis of Similarity (ANOSIM, a re-sampling test in which the null hypothesis is tested by comparing how often the real data produces more extreme differences that randomly resampled data, Primer 5.0, Primer-E Ltd., Quest Research Limited, Auckland, New Zealand) was used to test the null hypothesis that produce aggregations were similar between store classifications and the five classifications of percent white. We only analyzed percent white classification because our analysis of product richness showed percent white to be an important factor for produce diversity, but not median income. We performed the test using income classifications and the results were not meaningfully different than those generated using percent white classification. We performed a Similarity Percentage Breakdown Analysis (SIMPER, Primer 5.0, Primer-E Ltd., Quest Research Limited, Auckland, New Zealand) to determine which produce items contributed to similarities within groups and differences between groups.

This study was not subject to review and oversight by the Institutional Review Board because it did not involve human subjects.

## 3. Results

**Figure 2:**
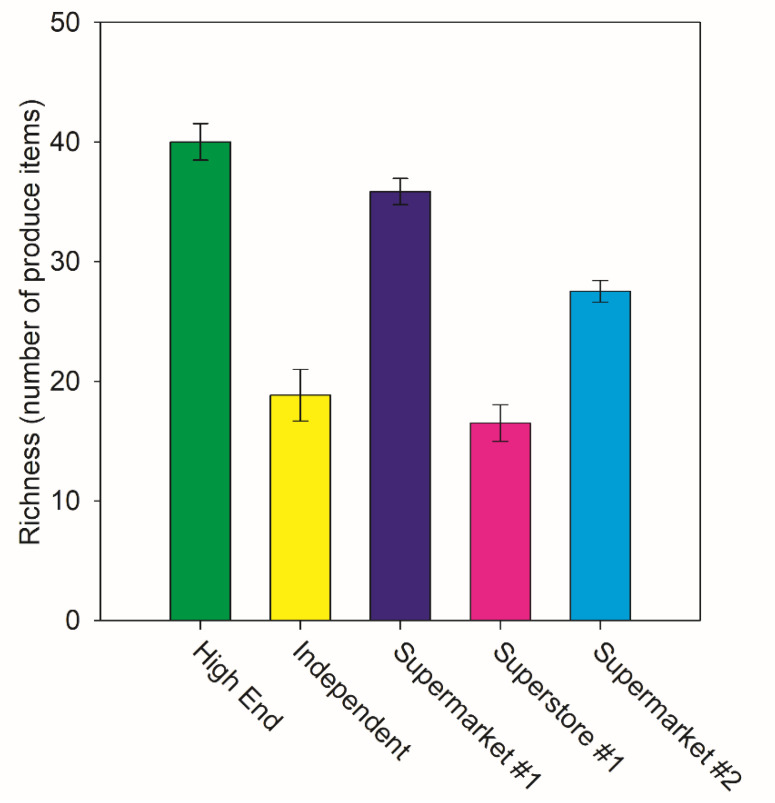
Species Richness for Each Market Class. Average species richness for Supermarket #1, Superstore #1, Supermarket #2, Independent stores, and High-end stores. Standard error bars included. Supermarket #1 and High-end stores had significantly greater species richness than Superstore #1 and independent stores.

**Figure 3:**
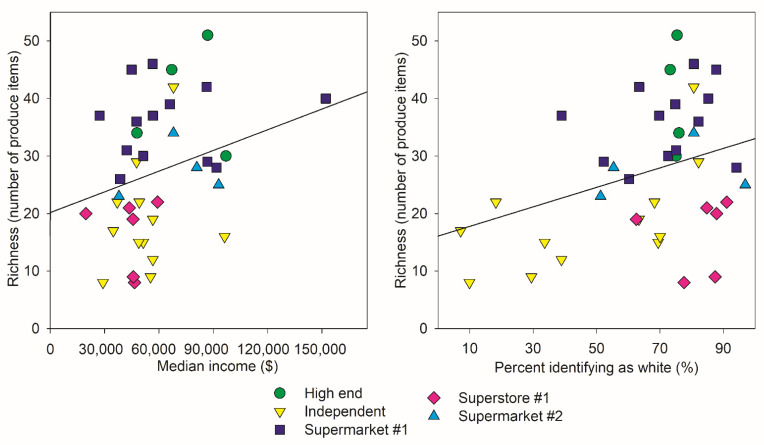
The relationship of Income and Percent White with Produce Diversity. Left: Median annual income of neighborhood plotted against species richness of the store. The line represents the outcome of the multiple regression (holding percent white constant). Right: Percent of population identifying as white plotted against species richness in store. The line represents the outcome of the multiple regression (holding median income constant).

The Analysis of Variance showed that species richness varied among store classifications but not income or whiteness category (Table 1 and Figure 2). The Tukey test indicated that “High-end” markets and Supermarket #1 had greater species richness than “Independent” markets and Superstore #1 by factor of two. Multiple regression revealed that the percentage of a neighborhood identifying as white had a parameter estimate of 0.1694 (*p* = 0.0378), but that the median income of the neighborhood’s parameter was not significantly different than zero (*p* = 0.1043). The adjusted R-square for the model was 0.1779, with total *p* = 0.0111 (Table 2 and Figure 3).

**Figure 4:**
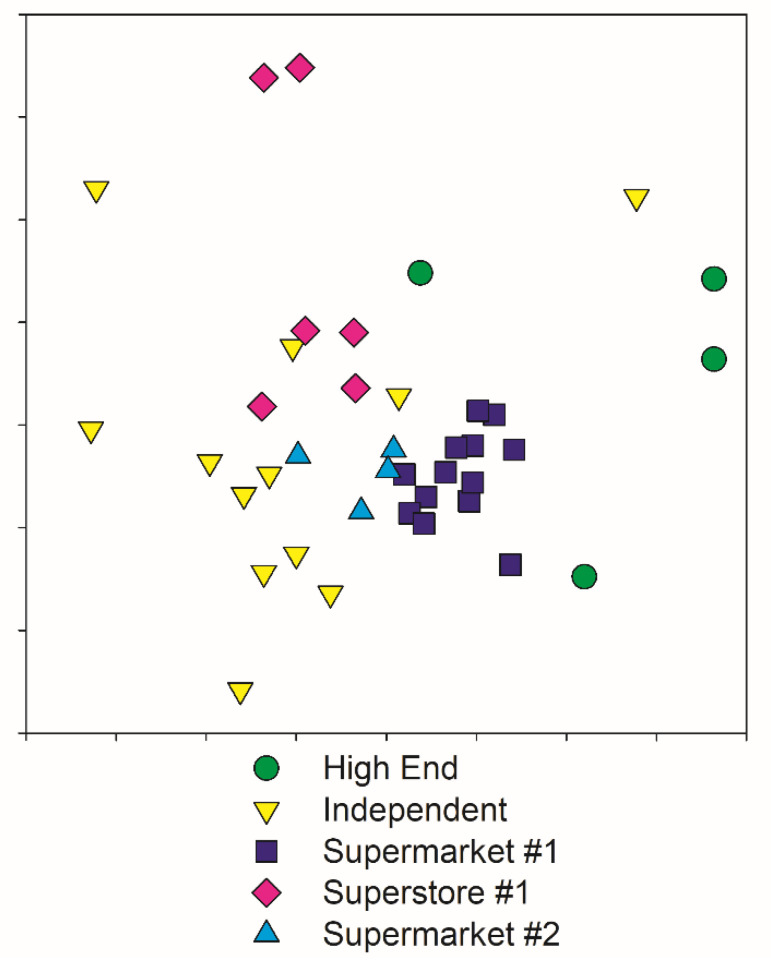
Multi-Dimensional Scaling (MDS) Ordination of Produce Similarity. MDS ordination representing the community at each supermarket. Bray–Curtis Dissimilarity values can be thought of as analogous to distance. The chart is generated by resampling multivariate space until a layout most closely representing the distances between all possible pairs of points is found. Thus distance between points is proportional to dissimilarity; the closer two points are, the more similar their assemblage of produce. Note that most store classification occupy their own distinct clusters, indicating greater similarity to each other than to the produce assemblages of others. The tighter clustering of stores like Supermarket #1 and Supermarket #2 indicate low variation between stores, whereas the larger spread of stores like Superstore #1 indicate less consistency in produce between locations. Stress (a measure of how well multivariate data is represented in two-dimensional space) for this ordination was 0.17, indicating a good representation of the data [24].

Analysis of Similarity showed that stores formed groups that were significantly different from each other (*p* = 0.004), but the classification of percent white did not (*p* = 0.761). Pairwise comparisons were performed (see Table 3). Independent stores and Supermarket #1 were significantly different from each other and the presence of Scotch bonnet peppers was the greatest contributor to their dissimilarity. Supermarket #1 and Superstore #1 were also significantly different from each other with broccoli as the greatest contributor to dissimilarity. All the other pairwise comparisons showed that assemblages were similar, except for High-end stores and Supermarket #2, which did not have enough replicates to compare. An MDS ordination allows visualization of each store’s relative similarity, in which points closer together have more similar communities of products and points farther apart have less similar communities (Figure 4). It is clear from the ordination that some store classifications, especially Winn-Dixie and Supermarket #1 form distinct clusters demonstrating low variability between locations, but others like the independent stores, High-end stores and Superstore #1 have greater differences in produce selection between locations, which is reflected in the greater distance between the points on the MDS ordination (Figure 4).

## 4. Discussion

Our analysis showed that different store classifications had different species richness of produce, but that those were not significantly related to the income or percent white categories that we used. However, using the numerical values we identified a pattern that showed that neighborhoods with a greater percentage identifying as white had on average greater produce species richness. Analysis of Similarity showed that the different store classifications had significantly different selections of produce available, and Similarity Percentage Breakdown Analysis identified some specific items that contributed to the small differences between the produce assemblages (Table 3). In contrast to our hypothesis, neither the neighborhood median income nor the percent of the population identifying as white significantly contributed to dissimilarity of the assemblages of produce available.

High-end markets had the highest average species richness of produce followed by Supermarket #1 (and they were not significantly different from each other). Independent stores and Superstore #1 had the lowest. Supermarket #2’s species richness was not significantly different from either the low or high extreme. Independent stores were highly variable, as might be expected since they are managed by different entities rather than large corporations. Regression analysis using continuous variables for percentage identifying as white and median income revealed that the percent of the population that was white was significantly associated with the product richness in a neighborhood, but median income did not which is shown in Figure 4. This may represent economic or environmental injustice against neighborhoods where a higher proportion of the population are minorities. Festival and Save-A-Lot had an average species richness of 8 compared to Presidente and Sedanos with an average species richness of 15.1. These independent stores showed such high variation in richness because of the manner in which we classified them. Some of them are more specialized serve Latinx customers, for example Presidente. Presidente had the highest abundance of bell peppers due to high consumer use (Figure 1). Others, like Lucky Market, cater more towards customers interested in organic produce or specialized produce for gourmet cooking. Some are found in poorer neighborhoods and just have fewer items available. These targeted customer groups demand different arrays of ingredients. Attempts to quantify produce diversity or to compare similarity of products available in different neighborhoods should expect patterns resulting from local demand owing to culturally specific cooking practices.

In the MDS ordination, the tight clustering of the points representing the produce assemblages found in Supermarket #1 (Figure 4) indicates low variability in stock between stores; Supermarket #2 was similarly tightly clustered, but with a smaller sample size. Independent stores varied in dissimilarity due to the variety of stores and their produce. The “High-end” stores were the least sampled due to fewer locations, related to their more expensive prices and general service to a higher income population. They carried a larger frequency of organic produce. High-end stores and Supermarket #1 had a significant difference in the array of produce available. Similarity percentage breakdown analysis showed that this difference was due to unique items, like Scotch bonnet peppers and organic items. Once again, independent stores were highly variable due to variety of communities that they served, variety of specialty items and whether they focused on higher-end or discount produce. Independent stores also had a significantly different array of produce than Supermarket #1 and Superstore #1.

Some limitations on the research were that markets were only visited on Thursday afternoons. The times that the market restocked the produce was not logged. Hurricane Dorian may have affected the stock of produce in districted examined during September and October 2019. The data collection focused on the type and amount of unpacked and refrigerated produce. Some produce like pumpkins and spaghetti squash were seasonal, which may have affected their availability during the data collection period. If this sample design of food desert study was applied to other geographical locations, there may be some limitations.

Long-term solutions need to be created to lessen the disparity of fresh produce available in neighborhoods of differing socio-economic characteristics. In the future, combining the ecological analysis presented in this paper with applications like Geographic Information Systems (GIS), can be used to identify, map and respond to food deserts as they appear. GIS has been applied in food desert research by visualizing food price index on a map to show areas of relative expensiveness, interpreting analysis of walking and public transit access to supermarkets, and creating maps showing the distance of stores selling food over a road network. Morton and Blanchard defined rural food deserts using GIS as counties where residents live more than 10 miles to the nearest grocery store [25]. The data can be organized to cross-examine whether the socioeconomic demographics of a neighborhood correlates to the placement of supermarkets. Communities and their leaders can use data like this to support establishment of markets carrying fresh, healthy food into neighborhoods where access to such food is needed.

Food deserts should not only be defined by geographic measures, but other influential factors like diversity and quality of food available. Previous food desert studies often involve lengthy interviews, and/or food index surveys, focus group discussions, administered consumer surveys, and an inventory of food [25]. Since food deserts may manifest in many ways, from effort to find food to availability, this makes it difficult for sample designs to identify specific gaps in the availability of healthy, fresh food. This research presents a method that can be used to precisely define urban food deserts, the types of communities they affect and specific deficiencies in the food available (for example, when communities have lower diversity of produce available, when produce assemblage is dissimilar from other, more affluent neighborhoods or when specific items are not available). The United States Department of Agriculture (USDA) determines what areas make up a food desert with the use of census tracts and examining the distance that must be traveled to access grocery stores [1]. Examining the relationships between grocery stores and the communities is essential to better define and understand how food deserts affect the health of these communities. Our work shows that multivariate statistics and community ecology statistics can be used to quantify food availability across communities and identify gaps in access to specific dietary needs. The method we use is relatively simple, easy to learn and implement and could be a powerful tool to improve public health. Further, this work could be easily adapted to train ecology students on diversity and similarity analyses in regions where natural environments are inaccessible.

## 5. Conclusions

In Broward County Florida, during the study period, we did not find *strong* differences in food availability for supermarkets in neighborhoods with different proportions of races and cultures or with relative wealth. However, we did find that the number of fresh produce products available was positively correlated with proportion identifying as white. This may represent environmental and/or social injustice with respect to community access to fresh fruits and vegetables.

These methods represent a new application of statistics that have been traditionally used in ecology. The methods demonstrate a technique to identify when the assemblages of products like fresh produce are dissimilar and less diverse in some communities. This is a quick and easy way to identify gaps in food availability in potentially marginalized communities, which opens an easier path to solving those problems.

## Figures and Tables

**Table 1:** Results from ANOVA (N = 39) performed using a General Linear Model (proc GLM, SAS 9.4, SAS Institute, Cary, NC, USA). The “*” indicates results significant at *p* < 0.05.

Source	Degrees of Freedom	Type III Sum of Squares	F Value	*p*
Store	4	1608.3	6.35	0.0175 *
Income class	4	191.4	0.76	0.5851
White class	4	481.1	1.9	0.2152
Store × Income class	2	2.6	0.02	0.9799
Store × White class	3	118.1	0.62	0.6228
Income class × White class	3	122.7	0.65	0.6097

**Table 2:** Parameter estimates for multiple regression of income and percent white relationships to produce diversity (N = 39). The “*” indicates results significant at *p* < 0.05.

Variable	DF	Parameter Estimate	Standard Error	t Value	*p*
Intercept	1	8.94852	5.96096	1.5	0.142
Median income	1	0.00011833	7.1 × 10^−5^	1.67	0.1043
Percent white	1	0.16942	0.07854	2.16	0.0378 *

**Table 3:** Pairwise comparisons of similarity between store classifications. Pairs of store classifications that are significantly different noted with an “*”. The third column shows the most important product contributing to differences between the stores as indicated by SIMPER.

Store	*p*-Value	Most Important Product Differences
Independent, High-end	0.60	Organic fennel/Organic cucumber (tie)
Independent, Supermarket #1	0.006 *	Scotch bonnet pepper
Independent, Superstore #1	1.00	Artichoke
Independent, Supermarket #2	0.833	Butternut squash
High-end, Supermarket #1	0.257	Scotch bonnet pepper
High-end, Superstore #1	0.200	Organic red beet/Organic fennel/Organic Romaine Lettuce/Organic cucumber/Organic golden beat (5-way tie)
High-end, Supermarket #2	-	Leek/Butternut squash (tie)
Supermarket #1, Superstore #1	0.005 *	Broccoli
Supermarket #1, Supermarket #2	0.083	Scotch bonnet pepper
Superstore #1, Supermarket #2	0.417	Broccoli

## Data Availability

Data from this project is archived here: https://works.bepress.com/j-matthew-hoch/65/.
